# From heart to mind: Linking interoception, emotion, and theory of mind

**DOI:** 10.1016/j.cortex.2017.02.010

**Published:** 2017-08

**Authors:** Punit Shah, Caroline Catmur, Geoffrey Bird

**Affiliations:** a Department of Neuroimaging, Institute of Psychiatry, Psychology and Neuroscience, King's College London, University of London, London, SE5 8AF, United Kingdom; b Department of Psychological Sciences, Birkbeck College, University of London, London, WC1E 7HX, United Kingdom; c Department of Psychology, Anglia Ruskin University, Cambridge, CB1 1PT, United Kingdom; d Department of Psychology, Institute of Psychiatry, Psychology and Neuroscience, King’s College, London, London, SE5 8AF, United Kingdom; e MRC Social, Genetic and Developmental Psychiatry Centre, Institute of Psychiatry, Psychology and Neuroscience, King's College London, University of London, London, SE5 8AF, United Kingdom; f Institute of Cognitive Neuroscience, University College London, London, WC1N 3AR, United Kingdom

**Keywords:** Mentalizing, Theory of mind, Interoception, Cardiac perception, Emotion, Predictive coding, Insula, Alexithymia, Mindreading

Theory of Mind (ToM) is traditionally characterized as the ability to represent mental states. Such a characterization leaves little room for studying individual differences in ToM – individuals either can, or cannot, represent mental states – and this binary classification cannot quantify the subtle individual differences observed in typical and atypical populations. In recognition of this problem, attempts have been made to provide a more detailed characterization of the constituent psychological processes which support the representation of mental states (Happé et al., 2017, Schaafsma et al., 2015), and the neurocomputational principles underpinning ToM (Koster-Hale & Saxe, 2013), in order to identify the source of individual differences. A recent model is of interest as it forwards the novel argument that interoception, perception of the internal state of the body, is a fundamental component of ToM (Ondobaka, Kilner, & Friston, 2017). Here we report the first test of the link between interoception and ToM.

Ondobaka, Kilner and Friston's model (Ondobaka et al., 2017) draws on the ‘Predictive Coding’ framework, in which the brain generates hypotheses about the world and tests their predictive validity against incoming sensory evidence. Several models within this framework argue for a role for interoception in emotion understanding (Seth, 2013), but Ondobaka and colleagues (Ondobaka et al., 2017) propose that, as emotional and other interoceptive states (e.g., hunger) constrain hypotheses about an individual's mental states, interoception plays a fundamental role in ToM. Strong and weak versions of this hypothesis can be constructed, where the weak version suggests that emotional and other interoceptive states provide evidence to form or evaluate hypotheses about another's mental state. The strong version of the hypothesis suggests interoceptive information is necessary for the representation of mental states – the defining feature of ToM. We therefore tested whether interoceptive accuracy predicted performance on the representation of mental states in general, or only in those situations where understanding emotion was crucial for accurate mental state representation.

**Fig. 1:**
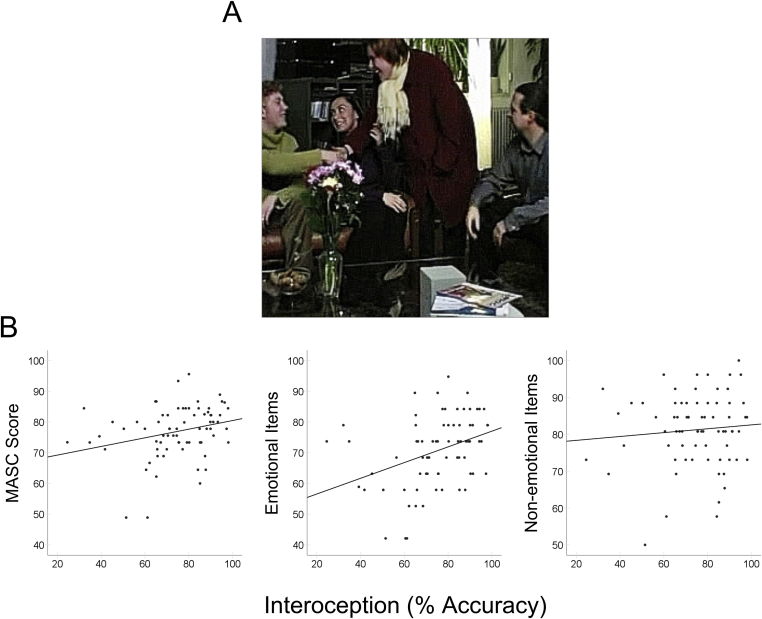
The link between interoception, emotion, and theory of mind. (A) The Movie for the Assessment of Social Cognition (MASC) was administered (Dziobek et al., 2006), in which participants watched a 15-min movie about a social interaction divided into short clips. After viewing each clip, they were presented with a multiple choice question requiring them to infer the mental state of one character. Only one of four answers was correct. Performance was quantified separately for emotional (e.g., “What is Sandra *feeling?*”) and non-emotional (e.g., “What is Michael *thinking?*”) questions. (B) Interoceptive accuracy was positively correlated with overall MASC score (*r*_*s*_ = .31, *P* = .008, left panel), driven by a significant association between interoception and emotional items (*r*_*s*_ = .41, *P* < .001, middle panel). However, there was no such association between interoception and the non-emotional items (*r*_*s*_ = .03, *P* = .80, right panel) and the two correlations were significantly different (*z* = 2.38, *P* = .017).

Seventy-two participants completed a well-established measure of interoception in which they counted their heartbeats during intervals of varying duration (Supplemental Experimental Procedures). They were not allowed to monitor their pulse by any means other than “silently concentrating on their heartbeats”. Each participant's heartbeat signals were recorded and, through comparison with their count, interoceptive accuracy was computed [see (Garfinkel, Seth, Barrett, Suzuki, & Critchley, 2015)]. Performance on this task may be influenced by one's ability to estimate time or count, so this was controlled for by measuring participants' ability to estimate time intervals of varying duration (Supplemental Experimental Procedures – Interoception and Time Estimation). Participants completed the Movie for the Assessment of Social Cognition (MASC), a well-validated measure of ToM [(Dziobek et al., 2006); see Supplemental Experimental Procedures], which required them to watch a social event in which accurate mental state inferences are needed to understand the story (Fig. 1A). The video was interspersed with multiple-choice questions probing mental state understanding from which an overall percentage accuracy score was derived. Accuracy was also computed for a set of nonsocial control questions (e.g., “What was the weather like on that evening?”). Most importantly, performance was quantified separately for questions which required representation of another's emotion (e.g., “What is Sandra *feeling?*”), and for those which did not require the representation of emotional states (e.g., “What is Michael *thinking?*”).

Greater interoceptive accuracy was associated with overall MASC score (*r*_*s*_ = .31, *P* = .008). Importantly, however, there was only a significant association between interoception and performance on items requiring the representation of another's emotion (*r*_*s*_ = .41, *P* < .001), not where representation of emotional states was not required (*r*_*s*_ = .03, *P* = .80). The size of these correlations was significantly different (*z* = 2.38, *P* = .017). This pattern of results (Fig. 1B) was supported by a Bayesian analysis and held after controlling for participants' age, gender, task completion time, time estimation ability and their performance on control questions (Supplemental Tables S1–S5).

Considerable efforts have been made to understand the biological basis of ToM, culminating in a wealth of data. There is also on-going debate about whether human and non-human animals have evolved a domain-specific module to represent mental states, or whether this process may be underpinned by domain-general mechanisms (Heyes, 2014). As long as the psychological and neural mechanisms supporting ToM are still to be determined such debate will continue. Understanding the neurocomputational principles supporting ToM is likely to provide a step-change in our ability to address these issues, and Predictive Coding models suggesting that interoception plays a role in social abilities contribute to this endeavor (Happé et al., 2017, Koster-Hale and Saxe, 2013, Ondobaka et al., 2017, Seth, 2013). The current results suggest that interoception is not necessary for the representation of mental states *per se*, however it contributes to accurate representation of mental states in situations where this process is reliant upon emotional, or otherwise interoceptive, information. It was also notable that performance on emotional questions (*M* = 70.53, *SD* = 11.56) was significantly (*t* = 7.41, *P* < .001, *d* = .06) worse than on non-emotional questions (*M* = 81.21, *SD* = 9.76), which may be due to the fact that emotional ToM requires processing of additional interoceptive information.

The current results are supported by evidence that insular cortex, known to be critical for generating interoceptive predictions, is a reliable neural correlate of affective processing (Bernhardt and Singer, 2012, Seth, 2013, Zaki et al., 2012). The findings are also in accordance with recent work showing that alexithymia, a condition characterized by interoceptive atypicalities (Hogeveen et al., 2016, Livingston and Livingston, 2016, Shah et al., 2016a, Shah et al., 2016b), predicted performance on a task requiring emotional understanding but not on a task assessing non-emotional ToM, whereas Autism Spectrum Disorder, which is associated with ToM but not interoceptive deficits, predicted performance on tests of ToM but not emotion understanding (Oakley, Brewer, Bird, & Catmur, 2016). Nonetheless, we suggest that interoceptive training may have a beneficial impact in the real world, where an improved ability to represent the interoceptive/emotional states of oneself and of others is likely to result in more accurate mental state inferences, and benefit emotional understanding more generally.

In sum, this study reports the first empirical test of Predictive Coding models of the contribution of interoception to ToM, and thereby i) speaks to the psychological and computational underpinnings of ToM and ii) provides impetus for future research on the basis of (atypical) ToM and related social abilities.
