# Supplementary material for: From heart to mind: Linking interoception, emotion, and theory of mind
Source: Cortex. 2017 Aug;93:220–3. doi: 10.1016/j.cortex.2017.02.010 (PMC5542037; doi:10.1016/j.cortex.2017.02.010)
Supplement: Supplementary file 1 [file mmc1.docx]

**Supplemental Experimental Procedures**

**Participants.** The sample size was determined prior to starting data collection. This was based on a power analysis which indicated that this sample size would be appropriate to detect a moderate sized correlation (where *r* = 0.40, power = 0.95, *α* = 0.05, two-tailed) between interoceptive accuracy and ToM. Participants were recruited using a local participant database, had normal or corrected-to-normal vision, and provided informed consent. Ethical clearance was granted by the local committee. Seventy-two adults (34 female) without a psychiatric diagnosis aged between 19 and 60 years (*Median*_age_ = 25.50, *M*_age_ = 28.9 years, *SD*_age_ = 9.27) participated in the study. One additional participant was tested, but later removed from analysis as they reported a psychiatric condition after completing the tasks. Another individual also participated but was later removed from analysis because they answered the questions (including non-social control questions) at random.

**Interoception and Time Estimation.** The Heartbeat Tracking task (Schandry, 1981) was used to measure interoception. Participants were seated in a quiet dimly-lit room and required to close their eyes and count their heartbeats during four intervals of varying duration (100, 35, 45, and 25 seconds). The order of intervals was randomized across participants. Heartbeat signals were acquired using a finger pulse oximeter (Contec Systems CMS-50D+; Qinhuangdao, China) attached to the non-dominant index finger, while the other arm was at rest. Participants were instructed not to monitor their heart by any means other than “silently concentrating on their heart beats”. Performance on the interoception measure may be influenced by one’s ability to estimate time, and neural correlates of interoception and time duration overlap in the insula (Wittmann, 2013). In the position described above, participants were therefore instructed to judge the duration of three randomized intervals (49, 37 and 19 seconds, e.g., Ainley, Brass, & Tsakiris, 2014). Interoception can be measured across other bodily axes (e.g., respiratory interoceptive accuracy; Garfinkel et al., 2016) and using other cardiac perception tasks (e.g., Whitehead, Drescher, Heiman, & Blackwell, 1977). We opted to use the Heartbeat Tracking task as it the most widely used interoception measure and it has found to be sensitive to individual differences (see Dunn et al., 2010).

Interoceptive Accuracy (see Garfinkel, Seth, Barrett, Suzuki, & Critchley, 2015) was calculated as performance on the Heartbeat Tracking task on a scale between 0 and 100% as follows: ¼ Σ(1-(|recorded number of heartbeats_interval_ – counted number of heartbeats_interval_|/recorded heart beats_interval_)) × 100. Higher scores indicate better interoceptive accuracy. Time Estimation ability was also computed as a percentage using this formula: ⅓ Σ(1-(|actual elapsed time_interval_ – estimated elapsed time_interval_|/actual elapsed time_interval_)) × 100. Higher scores indicate better time estimation ability.

**Movie for Assessment of Social Cognition.** The computerized Movie for Assessment of Social Cognition (MASC; Dziobek et al., 2006) was chosen because, unlike many ToM measures, it is sufficiently sensitive to individual differences in ToM ability. In this naturalistic task, participants watched a fifteen-minute movie divided into short clips about a group of 4 individuals at a social event. After viewing each clip, they were presented with a multiple choice question, requiring them to infer the mental state of one character (45 questions) or a nonsocial control question (5 questions) about the clip. Participants responded to each question by selecting from four possible answers and submitted their answer using a keypad. Only one of four answers was correct, hence performance on the MASC is indexed by a higher total score, which was converted into percentage accuracy. Performance was quantified separately for non-emotional ToM questions (e.g., “What is Michael *thinking?*”) and emotional questions (e.g., “What is Sandra *feeling?*”) as specified by Oakley, Brewer, Bird and Catmur (2016). There exist alternative ways to score the MASC which have previously been reported in the literature. Full details of the MASC, including theoretical considerations, its development, and technical specifications are reported in detail by Dziobek and colleagues (Dziobek et al., 2006, Montag et al., 2010; Montag et al., 2011; Preißler et al., 2010). The MASC was, in line with previous research, self-paced and administered on a computer screen, which took participants approximately 34 minutes (*M* = 33min 54 seconds, *SD* = 7 min 43 seconds) to complete. Task completion time was included in the analysis. The MASC, Interoception and Time Estimation tasks were administered such that the heartbeat tracking and time estimation tasks were completed by approximately half the sample before the MASC, while other participants completed the interoception and time estimation procedures after the MASC.

There were no significant outliers present in the data. However, a series of Kolmogorov-Smirnov tests showed that, apart from interoceptive accuracy (*p* = .17), the distribution of other variables (age, MASC scores, time estimation ability, task completion time, nonsocial control score) were significantly different from a normal distribution (all *p*s < .056). Non-parametric correlational analyses were therefore performed. In addition to the associations of main interest (reported in the main text), additional analyses indicated that interoception and time estimation were not associated (*r_s_* = 0.07, *p* = .53), and that overall MASC score was not associated with time estimation (*r_s_* = 0.03, *p* = .83) or the non-social control score (*r_s_* = 0.14, *p* = .23). There was no relationship between gender or task completion time and MASC score (*r_s_* = 0.11, *p* = .38 and *r_s_* = 0.02, *p* = .89, respectively). The correlations were similar when the data were tested separately for the emotional and non-emotional items and when Kendall’s tau-b correlational analyses were conducted (Correlation Matrix; Table S1).

Table S1

|  | |  | | **Interoceptive accuracy** | | | **Overall MASC score** | | **Emotional items** | | **Non-emotional items** | | **Control questions** | | **Completion Time** | | **Time estimation Ability** | **Age** | | | | **Gender** | |
| --- | --- | --- | --- | --- | --- | --- | --- | --- | --- | --- | --- | --- | --- | --- | --- | --- | --- | --- | --- | --- | --- | --- | --- |
| Interoceptive accuracy |  | Spearman's rho |  | | — |  | 0.308 | ** | 0.410 | *** | 0.030 |  | 0.107 |  | 0.220 |  | 0.074 | |  | -0.167 |  | -0.021 |  |
|  |  | p-value |  | | — |  | 0.008 |  | < .001 |  | 0.801 |  | 0.372 |  | 0.063 |  | 0.534 | |  | 0.161 |  | 0.863 |  |
|  |  | Kendall's tau B |  | | — |  | 0.215 | ** | 0.292 | *** | 0.022 |  | 0.086 |  | 0.148 |  | 0.051 | |  | -0.102 |  | -0.017 |  |
|  |  | p-value |  | | — |  | 0.010 |  | < .001 |  | 0.798 |  | 0.362 |  | 0.071 |  | 0.524 | |  | 0.216 |  | 0.861 |  |
| Overall MASC score |  | Spearman's rho |  | |  |  | — |  | 0.713 | *** | 0.781 | *** | 0.143 |  | 0.016 |  | 0.026 | |  | -0.236 | * | 0.105 |  |
|  |  | p-value |  | |  |  | — |  | < .001 |  | < .001 |  | 0.231 |  | 0.891 |  | 0.826 | |  | 0.046 |  | 0.381 |  |
|  |  | Kendall's tau B |  | |  |  | — |  | 0.571 | *** | 0.634 | *** | 0.117 |  | 0.008 |  | 0.022 | |  | -0.168 | * | 0.089 |  |
|  |  | p-value |  | |  |  | — |  | < .001 |  | < .001 |  | 0.231 |  | 0.922 |  | 0.788 | |  | 0.048 |  | 0.377 |  |
| Emotional items |  | Spearman's rho |  | |  |  |  |  | — |  | 0.246 | * | 0.064 |  | 0.192 |  | 0.057 | |  | -0.198 |  | 0.025 |  |
|  |  | p-value |  | |  |  |  |  | — |  | 0.037 |  | 0.594 |  | 0.106 |  | 0.637 | |  | 0.095 |  | 0.835 |  |
|  |  | Kendall's tau B |  | |  |  |  |  | — |  | 0.186 | * | 0.054 |  | 0.152 |  | 0.040 | |  | -0.140 |  | 0.022 |  |
|  |  | p-value |  | |  |  |  |  | — |  | 0.037 |  | 0.591 |  | 0.079 |  | 0.637 | |  | 0.109 |  | 0.833 |  |
| Non-emotional items |  | Spearman's rho |  | |  |  |  |  |  |  | — |  | 0.103 |  | -0.087 |  | -0.070 | |  | -0.131 |  | 0.151 |  |
|  |  | p-value |  | |  |  |  |  |  |  | — |  | 0.390 |  | 0.467 |  | 0.561 | |  | 0.273 |  | 0.207 |  |
|  |  | Kendall's tau B |  | |  |  |  |  |  |  | — |  | 0.083 |  | -0.067 |  | -0.045 | |  | -0.109 |  | 0.130 |  |
|  |  | p-value |  | |  |  |  |  |  |  | — |  | 0.405 |  | 0.437 |  | 0.599 | |  | 0.208 |  | 0.204 |  |
| Control questions |  | Spearman's rho |  | |  |  |  |  |  |  |  |  | — |  | -0.061 |  | -0.174 | |  | -0.260 | * | -0.028 |  |
|  |  | p-value |  | |  |  |  |  |  |  |  |  | — |  | 0.610 |  | 0.144 | |  | 0.027 |  | 0.813 |  |
|  |  | Kendall's tau B |  | |  |  |  |  |  |  |  |  | — |  | -0.050 |  | -0.134 | |  | -0.207 | * | -0.027 |  |
|  |  | p-value |  | |  |  |  |  |  |  |  |  | — |  | 0.602 |  | 0.158 | |  | 0.033 |  | 0.811 |  |
| Completion Time |  | Spearman's rho |  | |  |  |  |  |  |  |  |  |  |  | — |  | -0.039 | |  | 0.225 |  | -0.043 |  |
|  |  | p-value |  | |  |  |  |  |  |  |  |  |  |  | — |  | 0.745 | |  | 0.057 |  | 0.720 |  |
|  |  | Kendall's tau B |  | |  |  |  |  |  |  |  |  |  |  | — |  | -0.024 | |  | 0.152 |  | -0.036 |  |
|  |  | p-value |  | |  |  |  |  |  |  |  |  |  |  | — |  | 0.770 | |  | 0.071 |  | 0.718 |  |
| Time estimation ability |  | Spearman's rho |  | |  |  |  |  |  |  |  |  |  |  |  |  | — | |  | -0.163 |  | -0.044 |  |
|  |  | p-value |  | |  |  |  |  |  |  |  |  |  |  |  |  | — | |  | 0.170 |  | 0.712 |  |
|  |  | Kendall's tau B |  | |  |  |  |  |  |  |  |  |  |  |  |  | — | |  | -0.117 |  | -0.036 |  |
|  |  | p-value |  | |  |  |  |  |  |  |  |  |  |  |  |  | — | |  | 0.159 |  | 0.710 |  |
| Age |  | Spearman's rho |  | |  |  |  |  |  |  |  |  |  |  |  |  |  | |  | — |  | 0.048 |  |
|  |  | p-value |  | |  |  |  |  |  |  |  |  |  |  |  |  |  | |  | — |  | 0.686 |  |
|  |  | Kendall's tau B |  | |  |  |  |  |  |  |  |  |  |  |  |  |  | |  | — |  | 0.041 |  |
|  |  | p-value |  | |  |  |  |  |  |  |  |  |  |  |  |  |  | |  | — |  | 0.683 |  |
| Gender |  | Spearman's rho |  | |  |  |  |  |  |  |  |  |  |  |  |  |  | |  |  |  | — |  |
|  |  | p-value |  | |  |  |  |  |  |  |  |  |  |  |  |  |  | |  |  |  | — |  |
|  |  | Kendall's tau B |  | |  |  |  |  |  |  |  |  |  |  |  |  |  | |  |  |  | — |  |
|  |  | p-value |  | |  |  |  |  |  |  |  |  |  |  |  |  |  | |  |  |  | — |  |
|  | | | | | | | | | | | | | | | | | | | | | | | |
| **p* < .05, ** *p* < .01, *** *p* < .001 | | | | | | | | | | | | | | | | | | | | | | | |

Correlational data were also analyzed using JASP (https://jasp-stats.org; Love et al., 2015) to examine the strength of the evidence in favor of the null and experimental hypotheses. See Table S2 for a Bayes factors (BF10) matrix, where values > 3.0 and < 0.3 are taken as evidence in favor of the experimental and null hypotheses, respectively. In line with the aforementioned correlations, this demonstrates moderate evidence for the relationship between interoception and overall MASC score, very strong evidence for the link between interoception and emotional items, and moderate evidence *against* the association between interoception and non-emotional items on the MASC.

Table S2

|  | | | | | | | | | | | | | | |  |
| --- | --- | --- | --- | --- | --- | --- | --- | --- | --- | --- | --- | --- | --- | --- | --- |
|  |  | | **Interoceptive accuracy** | | | **Overall MASC Score** | | | **Emotional items** | | | **Non-emotional items** | | |  |
| Interoceptive Accuracy |  | Pearson's r |  | — |  | | 0.273 |  | | 0.365 | ** | | 0.089 |  | |
|  |  | BF₊₀ |  | — |  | | 4.076 |  | | 38.26 |  | | 0.295 |  | |
| Overall MASC Score |  | Pearson's r |  |  |  | | — |  | | 0.776 | *** | | 0.825 | *** | |
|  |  | BF₊₀ |  |  |  | | — |  | | 7.315e +22 |  | | 1.499e +33 |  | |
| Emotional items |  | Pearson's r |  |  |  | |  |  | | — |  | | 0.353 | * | |
|  |  | BF₊₀ |  |  |  | |  |  | | — |  | | 27.120 |  | |
| Non-emotional items |  | Pearson's r |  |  |  | |  |  | |  |  | | — |  | |
|  |  | BF₊₀ |  |  |  | |  |  | |  |  | | — |  | |
|  | | | | | | | | | | | | | | |  |
| *Note*. For all tests, the alternative hypothesis specifies that the correlation is positive. | | | | | | | | | | | | | | |  |
| * BF₊₀ > 10, ** BF₊₀ > 30, *** BF₊₀ > 100 | | | | | | | | | | | | | | |  |

Data were entered into a hierarchical regression, such that variables of no interest (age, gender, time estimation ability, non-social control question score, task completion time) were entered in the first and interoception entered in the second step. No variable in Step 1 predicted overall MASC score (all *p*s > .26). Importantly, when interoceptive accuracy was entered in Step 2, it was predictive of MASC score (*β* = 0.28, *t* = 2.36) leading to a significant change in *R*^2^ (*p* = .021), while other variables remained non-significant (other *p*s > .27).

When the equivalent analysis was performed with emotional questions entered as the dependent variable, a similar pattern of results emerged, whereby interoceptive accuracy was the only significant predictor (*β* = 0.34, *t* = 2.90, *p* = .005). In contrast, and following the correlational analyses, when non-emotional ToM performance was submitted as the dependent variable, no predictor variables, including interoceptive ability (*p =* .35), reached statistical significance (all other *p*s > 0.18).

Finally, the pattern of results (Tables S3 – S5) was similar when the data were entered into a Bayesian linear regression. There was moderate evidence for the association between interoceptive accuracy and overall MASC score and strong evidence for the link between interoception and emotional items. However, following the Bayesian correlations, there was in fact anecdotal evidence *against* the association between interoceptive accuracy and non-emotional items.

Table S3

| **Overall MASC Score** | | | | | | | | | | | |
| --- | --- | --- | --- | --- | --- | --- | --- | --- | --- | --- | --- |
| **Models** | | **P(M)** | | **P(M\|data)** | | **BF _M_** | | **BF _10_** | | **% error** | |
| Null model (incl. Age, Time Estimation Ability, Completion Time, Control Questions, Gender) |  | 0.500 |  | 0.203 |  | 0.255 |  | 1.000 |  |  |  |
| Interoceptive Accuracy |  | 0.500 |  | 0.797 |  | 3.922 |  | 3.922 |  | 0.002 |  |
|  | | | | | | | | | | | |
| *Note.*  All models include Age, Time Estimation Ability, Completion Time, Control Questions, Gender. | | | | | | | | | | | |

Table S4

| **Model Comparison - Emotional items** | | | | | | | | | | | |
| --- | --- | --- | --- | --- | --- | --- | --- | --- | --- | --- | --- |
| **Models** | | **P(M)** | | **P(M\|data)** | | **BF _M_** | | **BF _10_** | | **% error** | |
| Null model (incl. Age, Time Estimation Ability, Completion Time, Control Questions, Gender) |  | 0.500 |  | 0.086 |  | 0.094 |  | 1.000 |  |  |  |
| Interoceptive Accuracy |  | 0.500 |  | 0.914 |  | 10.611 |  | 10.611 |  | 0.010 |  |
|  | | | | | | | | | | | |
| *Note.*  All models include Age, Time Estimation Ability, Completion Time, Control Questions, Gender. | | | | | | | | | | | |

Table S5

| **Model Comparison - Non-emotional items** | | | | | | | | | | | |
| --- | --- | --- | --- | --- | --- | --- | --- | --- | --- | --- | --- |
| **Models** | | **P(M)** | | **P(M\|data)** | | **BF _M_** | | **BF _10_** | | **% error** | |
| Null model (incl. Age, Time Estimation Ability, Completion Time, Control Questions, Gender) |  | 0.500 |  | 0.586 |  | 1.414 |  | 1.000 |  |  |  |
| Interoceptive Accuracy |  | 0.500 |  | 0.414 |  | 0.707 |  | 0.707 |  | 0.002 |  |
|  | | | | | | | | | | | |
| *Note.*  All models include Age, Time Estimation Ability, Completion Time, Control Questions, Gender. | | | | | | | | | | | |

**Supplemental References**

Ainley, V., Brass, M., & Tsakiris, M. (2014). Heartfelt imitation: high interoceptive awareness is linked to greater automatic imitation. *Neuropsychologia*, *60*, 21-28.

Dunn, B. D., Galton, H. C., Morgan, R., Evans, D., Oliver, C., Meyer, M., ... & Dalgleish, T. (2010). Listening to your heart how interoception shapes emotion experience and intuitive decision making. *Psychological Science*, *21*(12), 1835-1844.

Dziobek, I., Fleck, S., Kalbe, E., Rogers, K., Hassenstab, J., Brand, M., ... & Convit, A. (2006). Introducing MASC: a movie for the assessment of social cognition. *Journal of Autism and Developmental Disorders*, *36*(5), 623-636.

Garfinkel, S. N., Manassei, M. F., Hamilton-Fletcher, G., den Bosch, Y. I., Critchley, H. D., & Engels, M. (2016). Interoceptive dimensions across cardiac and respiratory axes. *Philosophical Transactions of the Royal Society B*, *371*(1708), 20160014.

Garfinkel, S. N., Seth, A. K., Barrett, A. B., Suzuki, K., & Critchley, H. D. (2015). Knowing your own heart: distinguishing interoceptive accuracy from interoceptive awareness. *Biological Psychology*, *104*, 65-74.

Love, J., Selker, R., Marsman, M., Jamil, T., Dropmann, D., Verhagen, A. J., & Wagenmakers, E. J. (2015). JASP (Version 0.7.5.5) [computer software]. *Amsterdam. JASP Project*.

Montag, C., Dziobek, I., Richter, I. S., Neuhaus, K., Lehmann, A., Sylla, R., ... & Gallinat, J. (2011). Different aspects of theory of mind in paranoid schizophrenia: evidence from a video-based assessment. *Psychiatry Research*, *186*, 203-209.

Montag, C., Ehrlich, A., Neuhaus, K., Dziobek, I., Heekeren, H. R., Heinz, A., & Gallinat, J. (2010). Theory of mind impairments in euthymic bipolar patients. *Journal of Affective Disorders*, *123*, 264-269.

Oakley, B. F., Brewer, R., Bird, G., & Catmur, C. (2016). Theory of mind is not theory of emotion: A cautionary note on the Reading the Mind in the Eyes Test. *Journal of Abnormal Psychology*, *125*, 818-823.

Preißler, S., Dziobek, I., Ritter, K., Heekeren, H. R., & Roepke, S. (2010). Social cognition in borderline personality disorder: evidence for disturbed recognition of the emotions, thoughts, and intentions of others. *Frontiers in Behavioral Neuroscience*, *4*, 182.

Schandry, R. (1981). Heart beat perception and emotional experience. *Psychophysiology*, *18*, 483-488.

Whitehead, W. E., Drescher, V. M., Heiman, P., & Blackwell, B. (1977). Relation of heart rate control to heartbeat perception. *Biofeedback and Self-regulation*, *2*, 371-392.

Wittmann, M. (2013). The inner sense of time: how the brain creates a representation of duration. *Nature Reviews Neuroscience*, *14*, 217-223.
